# Early-onset neonatal hyperkalemia associated with maternal hypermagnesemia: a case report

**DOI:** 10.1186/s12887-018-1048-4

**Published:** 2018-02-13

**Authors:** Kenichi Tanaka, Hiroko Mori, Rieko Sakamoto, Shirou Matsumoto, Hiroshi Mitsubuchi, Kimitoshi Nakamura, Masanori Iwai

**Affiliations:** 1 Division of Neonatology, Kumamoto University Hospital, 1-1-1 Honjo, Chuo-ku, Kumamoto, 860-8556 Japan; 2 Department of Pediatrics, Graduate School of Life Science, Kumamoto University, 1-1-1 Honjo, Chuo-ku, Kumamoto, 860-8556 Japan

**Keywords:** Neonatal nonoliguric hyperkalemia, Progressive early-onset hyperkalemia, Maternal hypermagnesemia, Na^+^/K^+^-ATPase, Renal outer medullary potassium channel

## Abstract

**Background:**

Neonatal nonoliguric hyperkalemia (NOHK) is a metabolic abnormality that occurs in extremely premature neonates at approximately 24 h after birth and is mainly due to the immature functioning of the sodium (Na^+^)/potassium (K^+^) pump. Magnesium sulfate is frequently used in obstetrical practice to prevent preterm labor and to treat preeclampsia; this medication can also cause hypermagnesemia and hyperkalemia by a mechanism that is different from that of NOHK. Herein, we report the first case of very early-onset neonatal hyperkalemia induced by maternal hypermagnesemia.

**Case presentation:**

A neonate born at 32 weeks of gestation developed hyperkalemia (K^+^ 6.4 mmol/L) 2 h after birth. The neonate’s blood potassium concentration reached 7.0 mmol/L 4 h after birth, despite good urine output. The neonate and his mother had severe hypermagnesemia caused by intravenous infusion of magnesium sulfate given for tocolysis due to pre-term labor.

**Conclusion:**

The early-onset hyperkalemia may have been caused by the accumulation of potassium ions transported through the placenta, the shift of potassium ions from the intracellular to the extracellular space in the infant due to the malfunctioning of the Na^+^/K^+^ pump and the inhibition of renal distal tube potassium ion secretion, there is a possibility that these mechanisms were induced by maternal and fetal hypermagnesemia after maternal magnesium sulfate administration. Because neonatal hyperkalemia poses a significant risk for the development of life-threatening cardiac arrhythmia, this case highlights the necessity of maternal blood magnesium monitoring during magnesium sulfate administration and neonatal blood potassium monitoring when there is severe maternal hypermagnesemia at delivery.

## Background

Neonatal nonoliguric hyperkalemia (NOHK) is a frequently observed electrolyte imbalance that occurs in nonoliguric premature infants during the first days after birth [1]. NOHK results secondary to potassium shift from the intracellular space to the extracellular space partly because of the malfunctioning of the Na^+^/K^+^ pump due to the immature activity of Na^+^/K^+^-ATPase [2]. It can cause life-threatening cardiac arrhythmia as a severe complication [3].

Magnesium is a modulator of the Na^+^ and K^+^ ion transport systems in numerous tissues, and hypermagnesemia inhibits K^+^ ion transport from the extracellular to the intracellular space through the Na^+^/K^+^ pump [4]. Additionally, hypermagnesemia inhibits renal distal tube K^+^ ion secretion by the renal outer medullary K^+^ (ROMK) channel, which is an inward-rectifying K^+^ ion channel responsible for basal K^+^ ion secretion [5, 6]. An overdose of magnesium sulfate, frequently used in obstetrical practice for the prevention of preterm labor and to treat preeclampsia, can cause maternal hypermagnesemia [7]. Because the magnesium ion administered to the mother readily crosses the placenta, infants born to mothers with hypermagnesemia often develop transient hypermagnesemia during the first days after birth [8]. Moreover, it has been reported maternal and neonatal magnesium concentrations were highly correlated [9].

Although transient hyperkalemia during magnesium sulfate therapy in two pregnant drug abusers has been reported [7], there has been no report on neonatal transient hyperkalemia caused by maternal magnesium sulfate therapy. Here, we present the case of a male infant at 32 weeks gestation (weight 1268 g) that developed hyperkalemia immediately after birth due to neonatal and maternal transient hypermagnesemia after administration of magnesium sulfate.

## Case presentation

A 29-year-old Japanese woman in her second pregnancy was being treated for pregnancy-induced hypertension and preeclampsia with a drip infusion of magnesium sulfate at 30 weeks 2 days gestation. The dose of continuously infused magnesium sulfate was 0.1 g/h on day 1, 0.5 g/h on day 2–5, and 1.0 g/h on day 6–12. The dose was increased to 2.0 g/h to control an imminent preterm delivery on day 12, just before an emergency transfer to our hospital. During this treatment, maternal serum magnesium levels were not monitored. The mother had no medical conditions or any intake of other medications within these 12 days, which could have influenced blood potassium concentration.

On admission to our hospital, the patient could not mobilize independently because of hypotonia caused by severe hypermagnesemia and hypocalcemia (Table 1). On the same day, she delivered a male infant through vaginal birth. Her laboratory analysis at 7 h before and 7 h after delivery showed that the serum concentration of potassium, sodium, and creatinine ranged from 4.3 to 6.0 mmol/L, 133 to 128 mmol/L, and 0.85 to 0.95 mg/dL, respectively. These levels returned to within normal ranges at 5 days after delivery.

**Table 1:** The progression of blood and urine laboratory data of the mother and the infant

	Mother			Infant			
Sampling time	Day of delivery(7 h before delivery)	1 day after delivery(7 h after delivery)	5 days after delivery (at discharge)	Day of birth	1 day after birth	2 days after birth	30 days after birth
Mg (mg/dL)	9.9	5.9	–	8.7	7.7	5.9	2.0
Ca (mg/dL)	6.3	6.2	8.0	8.5	8.0	8.8	9.7
P (mg/dL)	–	–	–	10.4	8.7	6.2	6.9
K (mmol/L)	4.3	6.0	4.8	6.4	6.0	5.2	3.4
Na (mmol/L)	133	128	138	131	136	139	140
Cre (mg/dL)	0.85	0.95	0.56	0.82	1.07	1.10	0.32
BUN (mg/dL)	20.3	25.7	14.1	22.2	25.0	19.8	3.5
Urine K (mmol/L)	–	–	–	13	16	3	–
FeK (%)	–	–	–	36.5	24.9	4.4	–

*Mg* serum magnesium concentration, *Ca* serum calcium concentration, *K* serum potassium concentration, *Na* serum sodium concentration, *Cre* serum creatinine concentration, *BUN* Blood urea nitrogen, serum, *FeK* Fractional excretion of potassium, *Urine K* Urine potassium concentration (a spot urine specimen)

The male infant was born at 32 weeks gestation weighing 1268 g and with Apgar scores of 8 at 1 min and 9 at 5 min. He was immediately admitted to the neonatal intensive care unit in our hospital. There was no evidence of respiratory distress syndrome on chest radiograph, and he had a stable microbubble test. His heart rate was 130 beats per minute, and the arterial blood pressure was 42/22 mmHg, with normal contraction of the left ventricle confirmed by echocardiographic examination. His muscle tone was determined to be within the normal range by two expert neonatologists. The size and shape of both kidneys on ultrasonography were within the normal range, and his first urine was observed at 2 h after birth.

**Fig. 1:**
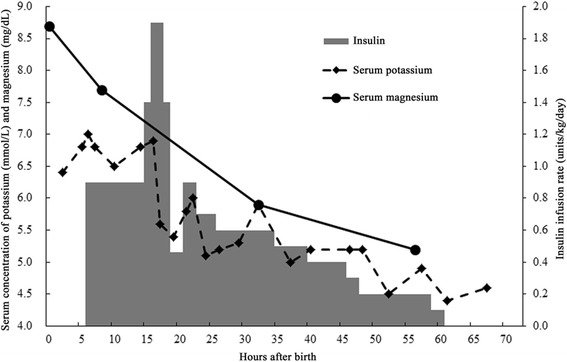
The neonate’s clinical course until 72 h after birth. The clinical course of the patient is shown. The main indicators include the serum potassium and magnesium concentrations and infusion rate of insulin until 72 h after birth

Laboratory data showed that his venous blood potassium concentration was 6.4 mmol/L at 2 h after birth and reached 7.0 mmol/L at 4 h after birth even though he had sufficient urine output (Table 1, Fig. 1). Glucose-insulin combined therapy was administered for 54 h with an insulin infusion rate between 0.9 units/kg/day and 1.9 units/kg/day to maintain his serum potassium concentration below 6.0 mmol/L. All blood samples were obtained from a catheter inserted directly into the right radial artery. During the therapy, he was not given potassium. His urine output was 4.1 mL/kg/h during the first 8 h, 6.2 mL/kg/h during the next day, and 3.3 mL/kg/h during the third day after birth with insufficient urinary potassium excretion (Table 1).

Laboratory analysis of his umbilical blood also showed hypermagnesemia at birth, which gradually subsided (Table 1, Fig. 1). The serum calcium concentration remained over 7.3 mg/dL with intravenous drip administration of calcium sulfate. Although transient hyponatremia was observed at 6 h after birth, it recovered in 24 h without sodium administration.

After resolution of NOHK, the neonate’s serum concentrations of potassium and magnesium were within the normal range. He was discharged from our hospital at 72 days after birth with no clinical complications and had normal growth and development at 3 years of age.

## Discussion

NOHK is a metabolic disorder that usually occurs in extremely premature neonates; therefore, the onset at 32 weeks old in the neonate presented in this case is less expected. Furthermore, the clinical course in this case was very unique in terms of the early onset at 2 h after birth and prolonged for 54 h under glucose-insulin combined therapy.

One possible explanation as to the cause of the early onset hyperkalemia is maternal hyperkalemia caused by hypermagnesemia [7]. Usually, the fetal plasma K^+^ ion concentration is higher than the maternal plasma concentrations [10] because of active K^+^ ion transport across the placenta [11]. In this case, the maternal potassium concentration increased from 4.3 mmol/L at 7 h before delivery to 6.0 mmol/L at 7 h after delivery. It is therefore possible that the excess K^+^ ions might have crossed from the mother to the fetus through the placenta and might have partially contributed to early onset neonatal hyperkalemia.

Alternatively, the prolonged hyperkalemia may have been secondary to a shift in K^+^ ions from the intracellular to the extracellular space. In premature infants with NOHK, erythrocyte Na^+^/K^+^-ATPase activity is significantly lower and the malfunctioning of the Na^+^/K^+^ pump induces a K^+^ ion shift from the intracellular to the extracellular space [1, 2]. This case may illustrate that even in an infant at 32 weeks gestation, Na^+^/K^+^-ATPase activity may be immature and result in malfunctioning of the Na^+^/K^+^ pump. In addition to prematurity, neonatal hypermagnesemia may produce a K^+^ ion shift from the intracellular to the extracellular space because of the high concentration of magnesium ions, which modulates the Na^+^/K^+^ ion transport systems in numerous tissues and inhibits the Na^+^/K^+^ pump exchange [4].

An additional mechanism whereby neonatal hypermagnesemia results in hyperkalemia is through the inhibition of renal distal tubule K^+^ ion secretion. Magnesium is thought to inhibit renal distal tube K^+^ ion secretion by suppressing the ROMK channel, which is an inward-rectifying K^+^ ion channel responsible for basal K^+^ ion secretion [5, 6]. In the neonate described in this case, post-delivery urine output was sufficient; however, the fractional excretion of potassium (FeK) at 0, 1, and 2 days after birth was 36.5, 24.9, and 4.4%, respectively, and these levels of excretion are lower than the average FeK (40 ± 5%) in NOHK during the first few days after birth [12]. The amount of urinary excretion of potassium is larger in an infant with NOHK than an infant with normokalemia [13]. However, in this case, it is interesting that urinary excretion of potassium was suppressed despite hyperkalemia, because of hypermagnesemia.

Thus, this progressive early-onset hyperkalemia may be caused by maternal and fetal hypermagnesemia. The underlying mechanism of this hyperkalemia is mainly assumed to be secondary to hypermagnesemia and subsequent malfunctioning of the Na^+^/K^+^-ATPase and inhibition of secretion in the ROMK channel.

## Conclusion

Maternal and fetal hypermagnesemia can induce rapidly progressive hyperkalemia in neonates. Because hyperkalemic infants are at high risk of developing life-threatening cardiac arrhythmias, we highlight the necessity of maternal blood magnesium monitoring during intravenous infusion of magnesium sulfate for tocolysis, as well as neonatal blood potassium monitoring when there is maternal severe hypermagnesemia at delivery.

## Abbreviations

NOHK: Neonatal nonoliguric hyperkalemia
ROMK: Renal outer medullary K^+^

## References

[1] Sato K, Kondo T, Iwao H, Honda S, Ueda K (1995). Internal potassium shift in premature infants: cause of nonoliguric hyperkalemia. J Pediatr.

[2] Baumgart S, Oh W, Guignard JP, Baumgart S (2008). Acute problems of prematurity: balancing fluid volume and electrolyte replacements in very low birth weight (VLBW) and extremely low birth weight (ELBW) neonates. Nephrology and fluid/electrolyte physiology.

[3] Hu PS, Su BH, Peng CT, Tsai CH (1999). Glucose and insulin infusion versus kayexalate for the early treatment of non-oliguric hyperkalemia in very-low-birth-weight infants. Acta Paediatr Taiwan.

[4] Bara M, Guiet-Bara A, Durlach J (1993). Regulation of sodium and potassium pathways by magnesium in cell membranes. Magnes Res.

[5] Heller BI, Hammarsten JF, Stutzman FL (1953). Concerning the effects of magnesium sulfate on renal function, electrolyte excretion, and clearance of magnesium. J Clin Invest.

[6] Huang CL, Kuo E (2007). Mechanism of hypokalemia in magnesium deficiency. J Am Soc Nephrol.

[7] Spital A, Greenwell R (1991). Severe hyperkalemia during magnesium sulfate therapy in two pregnant drug abusers. South Med J.

[8] Rantonen T, Kääpä P, Jalonen J, Ekblad U, Peltola O, Välimäki I (2001). Antenatal magnesium sulphate exposure is associated with prolonged parathyroid hormone suppression in preterm neonates. Acta Paediatr.

[9] Sherwin CM, Balch A, Campbell SC, Fredrickson J, Clark EA, Varner M, et al. Maternal magnesium sulphate exposure predicts neonatal magnesium blood concentrations. Basic Clin Pharmacol Toxicol. 2014;114:318–22. 10.1111/bcpt.12166 PMC3949120 24164968

[10] Shennan DB, Boyd CA (1987). Ion transport by the placenta: a review of membrane transport systems. Biochim Biophys Acta.

[11] Ashoor IF, de Jesús-González N, Somers MJ, Chishti AS, Alam S, Kiessling SG (2014). Fluid and electrolyte physiology in the fetus and neonate. Kidney and urinary tract diseases in the newborn.

[12] Omar SA, DeCristofaro JD, Agarwal BI, LaGamma EF (2000). Effect of prenatal steroids on potassium balance in extremely low birth weight neonates. Pediatrics.

[13] Stefano JL, Norman ME, Morales MC, Goplerud JM, Mishra OP, Delivoria-Papadopoulos M (1993). Decreased erythrocyte Na+, K+ -ATPase activity associated with cellular potassium loss in extremely low birth weight infants with nonoliguric hyperkalemia. J Pediatr.

